# HighHydrostaticPressure-Assisted Curing of *Vanilla planifolia*: Effects on Moisture Loss, Phenolic Content, Enzyme Activities, and Antioxidant Capacity

**DOI:** 10.3390/foods15183321

**Published:** 2026-09-19

**Authors:** Jorge E. Navarro-Baez, Jorge Welti-Chanes, Zamantha Escobedo-Avellaneda

**Affiliations:** Escuela de Ingeniería y Ciencias, Tecnologico de Monterrey, Av. Eugenio Garza Sada 2501 Sur, Col. Tecnológico, Monterrey 64700, NL, Mexico; a00820540@tec.mx (J.E.N.-B.); jwelti@tec.mx (J.W.-C.)

**Keywords:** glucovanillin, vanillin, vanilla curing, high hydrostatic pressure, β-glucosidase, phenylalanine ammonia-lyase

## Abstract

*Vanilla planifolia* curing is a critical process for developing vanillin and other aroma compounds; however, conventional thermal scalding (CTS) used during the killing stage may reduce the enzymatic activity required for phenolic compound formation during subsequent curing stages. This study evaluated the effect of high hydrostatic pressure (HHP) as a non-thermal killing method. Green vanilla pods were subjected to HHP treatments at 20–600 MPa for either 5 s or 5 min and compared with CTS. Moisture content, β-glucosidase and phenylalanine ammonia-lyase activities, total phenolic content, glucovanillin, vanillin, and antioxidant capacity were monitored throughout 20 sweating–drying cycles. Initial moisture content was 92%, but after 20 curing cycles, most HHP and CTS treatments reached final contents of 19.7–27.8%, except 20 MPa/5 min, which retained 60.4%, indicating delayed dehydration. Selected HHP treatments produced up to a fivefold higher initial apparent β-glucosidase activity than scalding and maintained higher activity during some early curing cycles, promoting glucovanillin conversion to vanillin. PAL activity increased immediately after the killing treatments, reaching 32.43 µmol min^−1^ kg^−1^ db for CTS and comparable values in specific HHP treatments (20 MPa/5 s, 50 MPa/5 s, and 100 MPa/5 min), demonstrating that PAL activity is affected by the pressure treatments. Selected HHP treatments also exhibited significantly higher TPC and antioxidant capacity than CTS samples at specific curing stages. Furthermore, an inverse relationship between glucovanillin depletion and vanillin formation was observed, resulting in greater vanillin accumulation and lower losses during curing. These findings indicate that HHP is a promising non-thermal alternative to conventional scalding for improving vanilla bean quality and potentially shortening the curing process.

## 1. Introduction

*Vanilla planifolia* is an orchid from subtropical regions of Mexico and is widely used in the cosmetic, pharmaceutical, and food industries worldwide [1]. Natural vanilla is highly valued due to its complex aromatic profile, consisting of a mixture of phenolics and other aromatic compounds that cannot be replicated by synthetic vanilla substitutes [2]. Although the production and availability of natural vanilla depend on multiple agronomic, environmental, and socioeconomic factors, the postharvest curing process plays a critical role in the formation of its characteristic aroma. Curing conditions strongly influence the hydrolysis of glucovanillin and the subsequent accumulation of vanillin, the major aroma compound in cured vanilla [3]. Therefore, optimizing vanilla curing remains a significant challenge for improving the yield and quality of vanillin and other vanilla compounds.

When vanilla pods are harvested, they exhibit a green color and lack the characteristic aroma associated with cured vanilla; the development of aroma, flavor, and the characteristic dark-brown color occurs during the curing process, which can take several weeks, involving complex biochemical and physiological transformations [4]. Traditional vanilla curing consists of four main stages: killing, sweating, drying, and conditioning [5]. The second and third stages are performed through multiple sweating–drying cycles to develop flavor and reduce water content. Killing is considered the most important stage; it aims to stop vegetative growth and disrupt cellular compartmentalization, allowing glycosylated phenolics (aroma precursors) to interact with hydrolytic enzymes (β-glucosidase) to initiate biochemical reactions responsible for aroma formation [6].

Glucovanillin is the most abundant and important glycosylated precursor of vanillin, the main aroma compound in vanilla which is released through the enzymatic hydrolysis of glucovanillin due to β-glucosidase [7]. Glucovanillin and β-glucosidase are compartmentalized within different cellular structures. Glucovanillin is most likely to be in the vacuole while β-glucosidase is in the cytoplasm of the vanilla cells [8]; therefore, the disruption of cell membranes during the killing stage is essential to promote enzymatic hydrolysis [9,10]. Prolonged β-glucosidase activity during curing is a key factor determining vanillin accumulation and overall vanilla quality [9].

Traditionally, the killing stage is performed by scalding in hot water at temperatures ranging from 60 to 100 °C for a short period [11,12], but while it is effective in the disruption of cellular structures, the high temperatures significantly reduce β-glucosidase activity [9,10]. Therefore, the development and application of alternative killing methods are essential to preserve enzyme activity. High hydrostatic pressure (HHP) is a non-thermal technology widely used for food preservation, capable of modifying cellular structures and enzyme activity without significantly altering nutritional or sensory attributes [13]. Depending on the intensity of the pressure, it can modify plant microstructure without inhibiting enzymes and may even enhance enzyme activity by inducing structural modifications [14,15]. In addition, HHP at moderate pressure may act as an abiotic stressor in plant tissues, potentially inducing metabolic responses associated with the synthesis of secondary metabolites such as phenolic compounds, since they play an important role in plant growth and development as a defense mechanism against abiotic stresses [13,16]. Phenylalanine ammonia lyase (PAL) is a key enzyme in the phenylpropanoid pathway and, as a response to stress conditions, may participate in the biosynthesis of phenolic compounds [17,18]. The activation of PAL has been associated with increased accumulation of phenolic compounds and enhanced antioxidant capacity in plant tissues subjected to abiotic stress [19]. However, the response of plant tissues to HHP depends strongly on treatment intensity; changes in phenolic concentration may also be attributed to cellular decompartmentalization or increased compound extractability [13]. Pressure-induced conformational changes may preserve or enhance enzymatic functionality or, under more severe conditions, lead to enzyme inactivation [14].

HHP has been previously studied as a killing method during vanilla curing at pressures from 100–400 MPa for 5 min and 50–600 MPa for 5 s at a controlled temperature (7 °C), evaluating phenolic compounds, color parameters, and β-D-glucosidase, polyphenol oxidase, and peroxidase activities [20,21]. Nevertheless, it has been demonstrated that the effects of HHP are highly dependent on processing temperature. So, the present study evaluated HHP treatment at room temperature with subsequent increments due to adiabatic compression. PAL activity, antioxidant capacity by ORAC, and moisture content were incorporated. Moisture content, β-glucosidase and PAL activities, total phenolic content, glucovanillin, vanillin, and antioxidant capacity were monitored within the same experimental framework (20–600 MPa for 5 s or 5 min) throughout the sweating–drying curing process. In addition, kinetic modeling was incorporated to describe moisture and glucovanillin loss throughout curing. Therefore, the present study provides an integrated analysis of the biochemical and physicochemical evolution of HHP-treated vanilla, offering insights into the response of vanilla beans during HHP-assisted curing at room temperature.

## 2. Materials and Methods

### 2.1. Chemicals

Sodium phosphate dibasic dodecahydrate, sodium phosphate monobasic hydrate, p-nitrophenyl β-D-glucopyranoside (p-NPG), boric acid, sodium hydroxide, polyvinylpolypyrrolidone (PVPP), β-mercaptoethanol, L-phenylalanine (L-Phe), glacial acetic acid, ethyl acetate, Folin–Ciocalteu reagent (2 N), Trolox (6-hydroxy-2,5,7,8-tetramethylchroman-2-carboxylic acid; TE), fluorescein sodium salt, 2,2′-azobis(2-amidinopropane) dihydrochloride (AAPH), gallic acid (GA), and vanillin standard (99% purity) were obtained from Sigma-Aldrich Corp (St. Louis, MO, USA). Formic acid, methanol, water, acetonitrile (used for chromatographic analyses were HPLC-grade), and ethanol were obtained from J.T. Baker (Center Valley, PA, USA). Unless otherwise specified, reagents were of analytical grade.

### 2.2. Plant Material

About 20 kg of vanilla beans (*Vanilla planifolia*), harvested in December 2022 from Papantla, Veracruz, Mexico (20°26′48″ N, 97°19′30″ W, 186 m above sea level), was used in this study. The pods were harvested at physiological maturity, when they presented a green color and the tip of pods (distal end) showed a yellowish tone. After harvesting, the pods were transported by land, requiring one day to reach the processing facilities (Emerging Technologies Laboratory at Tecnológico de Monterrey, Monterrey, Mexico), where they were processed the day following their arrival. Pods measuring 15–17 cm in length, with a diameter of 9–12 mm at the center of the pod and weighing 10–12 g per pod, were selected. Pods with visible defects or a curved shape were excluded, as curved shape and longer lengths prevented them from being inserted into the treatment chamber of the equipment.

### 2.3. Vanilla Curing

Vanilla pods were washed carefully using tap water. A group of 20 vanilla pods were separated and labelled as untreated fresh vanilla pods (UFVP), which were used exclusively as the untreated baseline reference and were not evaluated during subsequent curing cycles; additionally, approximately 50 beans were assigned to each treatment from the single harvest batch collected.

For the conventional scalding control, fresh vanilla pods were immersed in boiling water for 8 s to simulate the traditional killing method. For HHP treatment, samples were first placed in 25 × 36 cm polyethylene bags (Filmpack, S.A. de C.V., Monterrey, Mexico), vacuum-sealed (Torrey EVD-4, Mexico) and treated in AVURE 2L equipment (Columbus, OH, USA) using water at room temperature (22.9 ± 2.12 °C) as a pressure-transmitting medium [22]. Beans were treated at 20, 50, 100, 200, and 600 MPa for holding times of 5 min and 5 s, after the come-up time (CUT). For each treatment, the CUT required to reach the target pressure and the average chamber temperature during the holding period were recorded (the temperature was recorded in 5-s intervals, so an average value was reported), as shown in Table 1. Depending on the pressure level, CUT values ranged from approximately 10 to 90 s. Likewise, the average process temperature increased with pressure due to adiabatic compression, ranging from 23.1 °C in the lowest-pressure treatments to 38.9 °C in the highest-pressure treatment; no active temperature control was applied to compensate for adiabatic heating during pressurization. Depressurization was performed immediately after completion of the holding time.

After scalding and HHP treatment, vanilla pods were placed into a climatic chamber (POL-EKO KK400 TOP+/FIT P, Wodzisław Śląski, Poland), which was configured at 45 °C and a relative humidity (RH) > 90% during 19 h for the sweating step and 50 °C at a controlled RH of 60% and maximum light intensity of 15,000 lx during 5 h for drying. Samples for each treatment were randomly and homogenously distributed into the two trays of the climatic chamber. These conditions were applied repeatedly to complete 20 sweating–drying cycles, each lasting 24 h. Anywhere from 10 to 12 pods were sampled at cycles 0, 1, and 3, while at curing cycles 5, 12, and 20, 5–6 pods were sampled. Cycle 0 corresponded to samples collected immediately after the killing treatment, before placement in the climatic chamber.

### 2.4. Vanilla Storage and Homogenization

Immediately after completing the corresponding curing cycle, vanilla pods were taken to be frozen in liquid nitrogen. The samples were subsequently stored at −80 °C (Ultra-Low Temperature Freezer (PHCBI- VIP + PLUS, Wood Dale, IL, USA)) until analysis.

For subsequent analyses, the vanilla pods were homogenized to obtain a representative and uniform sample and to minimize potential sampling bias associated with differences in pod individual characteristics. Four to five pods from each treatment were pooled and ground in a food processor (Oster Xpert Series, Oster, Ciudad Acuña, México) for approximately 15 s until a homogeneous vanilla sample was obtained. The homogenized samples were used for β-glucosidase and phenylalanine ammonia-lyase activity assays. For the remaining analyses, including those of total phenolic content, glucovanillin and vanillin contents, and antioxidant activity, the homogenized vanilla samples were freeze-dried before analysis, as described in the following section.

### 2.5. Freeze Drying of Vanilla

Previously homogenized frozen samples corresponding to each treatment and curing cycle were dried in a freeze-dryer chamber (Labconco™ FreeZone™ Triad™ Freeze-Dry Systems, ES, Labconco Corporation, Kansas City, MO, USA). To allow all treatment samples corresponding to the same curing cycle to be processed simultaneously, freeze-drying of all samples was completed in six separate runs. The freeze-drying process was carried out at −55 °C and 0.04 mbar and terminated after the established 7-day processing period. After completion of the process, the freeze-dried vanilla samples were transferred to vacuum-sealed polyethylene bags and stored at −80 °C until further analysis.

### 2.6. Moisture Content in Fresh and Freeze-Dried Vanilla

Moisture content was determined gravimetrically [22] in a technical triplicate using the same homogenized sample. Approximately 1 g of homogenized sample was weighed in technical triplicate and dried in an oven at 80 °C for a fixed period of 24 h. This drying time had been previously verified to be sufficient to achieve constant weight under the conditions used. Residual moisture in freeze-dried samples was determined to correct the analytical results obtained from lyophilized material and express them on a dry-mass basis. Moisture content was calculated from the mass loss relative to the initial sample mass and expressed on a wet basis: Moisture content (%) = [(initial mass − dry mass)/initial mass] × 100.

### 2.7. β-Glucosidase Activity

β-glucosidase activity was analyzed as described by Buitimea-Cantúa et al. (2022) [20] with modifications. About 100 mg of homogenized fresh vanilla sample was placed into a mortar with 10 mL of 0.1 M phosphate buffer (pH 7.0). The sample was crushed, and the mixture was then centrifuged (1764× *g*/10 min/25 °C) to sediment the plant material. Subsequently, 2 mL of the supernatant was centrifuged again (14,196× *g*/5 min/4 °C) to remove any remaining suspended particles and obtain a clarified enzymatic extract.

From each enzymatic extract, three 100 µL aliquots were independently assayed in different wells of a flat-bottom, clear 96-well microplate with 100 µL of 0.04 M p-NPG as a substrate; therefore, β-glucosidase activity was measured in technical triplicate. As a blank, 100 µL of phosphate buffer and 100 µL of the substrate were used. Absorbance was read at 405 nm and 40 °C for 1 h with 1-min intervals using a plate reader (Synergy™ HTX, BioTek ^®^ Instruments, Inc., Winooski, VT, USA).

The enzymatic activity of β-glucosidase was calculated using the Beer–Lambert law, where p-nitrophenol formation was quantified using the molar extinction coefficient (18,500 M^−1^ cm^−1^) and the effective optical path length of 0.622 cm, from a 200 µL reaction volume. Blank absorbance was subtracted from the corresponding sample absorbance before calculating the reaction rate. The reaction rate was obtained from the slope of the linear portion of the absorbance-versus-time curve. Initial lag points and the final plateau region were excluded from the regression. For each curing cycle, a common time interval providing a coefficient of determination (R^2^) ≥ 0.90 was selected and applied consistently to all treatments within that cycle. The results were expressed as µmol of p-nitrophenol min^−1^ g^−1^ of sample on a dry basis (µmol min^−1^ g^−1^ db).

### 2.8. PAL Activity

PAL activity was analyzed as proposed by Ortega-Hernández et al. (2019) [23]. Homogenized frozen fresh vanilla samples were used for PAL extraction. About 2 g of the fresh vanilla sample was mixed with 8 mL of 50 mM borate buffer (pH 8.5) containing 5 g of PVPP per liter of buffer and 500 µL of β-mercaptoethanol per liter of buffer; this mixture was homogenized in a mortar for 1 min and then centrifuged (1764× *g*/5 min/25 °C) to separate most solids. A supernatant of 2 mL was centrifuged again (14,196× *g*/20 min/4 °C) to separate any remaining solids; this supernatant was considered the enzymatic extract.

The enzymatic reaction consisted of 240 µL of the enzymatic extract, 500 µL of 50 mM borate buffer (pH 8.5) without PVPP or β-mercaptoethanol, and 105 µL of 100 mM L-phenylalanine in a 2 mL microcentrifuge tube. For the blank reactions, L-phenylalanine was replaced with 50 mM borate buffer (pH 8.5) without PVPP or β-mercaptoethanol, for a total of 605 µL. The mixture was incubated at 37 °C for 1 h, and the reaction was stopped by adding 105 µL of glacial acetic acid. For each enzyme extract, five independent reaction mixtures were prepared from the same extract: three containing L-phenylalanine as technical replicates for enzyme activity determination and two corresponding blank reactions.

To extract the cinnamic acid produced during the reaction, 750 µL of ethyl acetate was added to the previously incubated mixture and vortex-mixed vigorously before centrifugation (14,196× *g*/5 min). An aliquot of 550 µL of the organic phase, which contains cinnamic acid, was dried in a vacuum concentrator (CentriVap^®^ Concentrator, Labconco Corporation, Kansas City, MO, USA) at 40 °C for 1 h.

To analyze the cinnamic acid content, the evaporated extract was dissolved in 150 µL of Methanol/Water (50/50, *v*/*v*), HPLC grade, and filtered through a 0.45 µm PTFE membrane. The content was quantified by HPLC-DAD (1200 series, Agilent Technologies, Inc., Santa Clara, CA, USA) using a C18 Column (250 × 4.6 mm, 5 µm, 100 Å; Luna, Phenomenex, Inc., Torrance, CA, USA). The mobile phases used were (Phase A) water acidified to pH 4.5 with formic acid and (Phase B) Methanol/Acetonitrile (60/40 *v*/*v*). The gradient used was 0/100, 3/90, 10–15/75, 16/70, 25/25, 30/10, 32–34/0, and 35–40/100 (min/% phase A) at a flow rate of 0.6 mL min^−1^. Trans-cinnamic acid was identified at 280 nm based on its retention time and DAD spectra. Quantification was performed using a 5-point calibration curve ranging from 0.5 to 25 ppm (R^2^ = 0.9997). PAL activity was calculated from the amount of trans-cinnamic acid produced during the 60-min incubation period and divided by the incubation time (60 min); it was expressed as µmol of trans-cinnamic acid equivalent min^−1^ kg^−1^ on a dry basis (µmol min^−1^ kg^−1^ db).

### 2.9. Total Phenolic Content (TPC)

TPC was analyzed using the method described by Waterhouse (2003) [24] with modifications. About 100 mg of lyophilized vanilla was mixed with 10 mL of absolute ethanol and macerated for 24 h at room temperature under constant agitation at 250 rpm using an orbital shaker incubator (TE-4200, TECNAL^®^, Piracicaba, SP, Brazil). After maceration, solids were separated by centrifugation (1764× *g*/10 min/25 °C); then the supernatant was filtered using a 0.45 µm PTFE membrane. A total of 50 µL of phenolic extract was mixed with 650 µL of water, 50 µL of Folin–Ciocalteu reagent, and 250 µL of 0.5 M sodium carbonate. For each phenolic extract, three independent Folin–Ciocalteu reaction mixtures were prepared from aliquots of the same extract and analyzed as technical replicates. The mixtures were incubated at 37 °C for 2 h. After incubation, 200 µL of the phenolic mixture was transferred into a 96-well flat, clear microplate, and absorbance was read at 765 nm in a microplate reader (Synergy HT, BioTek Instruments, Bad Friedrichshall, Germany). A 7-point gallic acid (GA) calibration curve was prepared in the range of 0–300 ppm (R^2^ = 0.993). TPC was reported as mg of gallic acid equivalents g^−1^ of vanilla on a dry basis (mg g^−1^ db).

### 2.10. Vanillin and Glucovanillin Content

For the extraction and quantification of vanillin and glucovanillin, the methodology of Voisine et al. [25] with modifications was used. About 100 mg of lyophilized vanilla was mixed with 10 mL of absolute ethanol and macerated for 24 h at room temperature under constant agitation at 250 rpm using an orbital shaker incubator (TE-4200, TECNAL^®^, Piracicaba, SP, Brazil). Then, the mixtures were centrifuged in a benchtop centrifuge (Allegra^®^ X-12, Beckman Coulter, Inc., Brea, CA, USA) at 1764× *g* for 10 min at room temperature. From the same supernatant, three (1 mL) aliquots were independently filtered through 0.45 µm PTFE membranes and transferred into separate HPLC vials for analysis.

A 2 µL sample of each extract was analyzed using an HPLC-DAD system (1200 series, Agilent Technologies, Inc., Santa Clara, CA, USA) using a C18 Zorbax Eclipse column (150 mm × 3 mm, 3.5 µm particle size); the mobile phases used were water acidified with 0.1% formic acid (Phase A) and methanol (Phase B). Detection and quantification were performed at 275 nm, the flow rate was at 0.4 mL min^−1^, and the solvent gradient was 0–3/90, 3–30/90–80, 30–37/80 (min/%A).

Vanillin was identified by comparing retention times and UV spectra with the corresponding standard. A 5-point calibration curve ranging from 0.1 to 10 mM (R^2^ = 0.9995) of vanillin was used to quantify phenolics as mg of vanillin equivalent (VE) per g of sample on a dry basis (mg g^−1^ db).

### 2.11. Antioxidant Capacity

The oxygen radical absorbance capacity (ORAC) method was used to determine antioxidant activity as described by Prior et al. [26,27]. Approximately 100 mg of freeze-dried vanilla was mixed with 10 mL of absolute ethanol and macerated for 24 h at room temperature under constant agitation at 250 rpm using an orbital shaker incubator (TE-4200, TECNAL^®^, Piracicaba, SP, Brazil). The ethanolic extract of vanilla was used for the preparation of a 1:100 dilution with phosphate buffer (75 mM, pH 7.4). From each diluted extract, three 25 µL aliquots were transferred into separate wells of a black, round-bottom 96-well polystyrene microplate and analyzed as technical replicates. Six wells containing only phosphate buffer were included as blanks.

A microplate reader (Synergy HT, BioTek Instruments, Inc., Bad Friedrichshall, Germany) was configured to dispense 150 µL of 1 µM fluorescein followed by 25 µL of 153 mM AAPH after 30 min of incubation at 37 °C. Fluorescence was measured for 60 min at 2-min intervals at a stable temperature of 37 °C (Excitation wavelength: 485/20 nm and emission wavelength: 528/20 nm). A five-point Trolox calibration curve ranging from 25 to 100 µM (R^2^ = 0.99) was used to quantify the antioxidant capacity.

Due to the number of samples that could be analyzed simultaneously, samples from each curing cycle were analyzed in a separate analytical run. Therefore, six ORAC runs were performed, corresponding to curing cycles 0, 1, 3, 5, 12, and 20. Each run included the treatments corresponding to the same curing cycle.

The area under the fluorescence decay curve (AUC) was calculated from the fluorescence measurements recorded during the 60-min assay. The AUC values obtained for the Trolox standards were used to generate the calibration curve relating antioxidant response to Trolox concentration. The Trolox-equivalent concentration obtained from the calibration curve was subsequently corrected using the corresponding dilution factor, the extraction volume (10 mL), and the dry mass of vanilla used for extraction. Antioxidant capacity was expressed as µmol Trolox equivalents (TE) g^−1^ db on a dry basis (µmol TE g^−1^ db).

### 2.12. Kinetic Modeling

The Weibull model was used to describe the moisture content of vanilla during curing cycles. The model was expressed as $M_{t}/M_{0}={\mathrm{e}}^{-{\left(t,/,\lambda \right)}^{k}}$, where *M_t_* and *M_0_* represent the moisture content (% wet basis) at the time of curing time (*t*) and at the beginning of the curing process (cycle 0) respectively; *t* is the curing time (days); *λ* is the scale parameter (days); and *k* is the dimensionless shape parameter. Model parameters were estimated by nonlinear least-squares regression by minimizing the sum of squared residuals between experimental and predicted moisture contents. The coefficient of determination (R^2^) was calculated on the original moisture-content scale as $R^{2}=1-\sum {\left(M_{exp}-M_{pred}\right)}^{2}/\sum {\left(M_{exp}-{\bar{M}}_{exp\;}\right)}^{2}$.

The decrease in glucovanillin content was described using a pseudo-first-order kinetic model expressed as $\ln\left[\left(C_{t}-C_{f}\right),/,\left(C_{i}-C_{f}\right)\right]=\;-kt$ where $C_{i}$, $C_{t}$ and $C_{f}$ represent the initial glucovanillin concentration, the concentration at a given curing cycle and the final concentration, respectively. The kinetic constant (k) was obtained from the slope of the linear regression of $\ln\left[\left(C-C_{f}\right),/,\left(C_{i}-C_{f}\right)\right]$ versus *t.* Predicted glucovanillin concentrations were calculated from the fitted kinetic model and compared with the experimental values.

In addition to the coefficient of determination of R^2^, model performance was evaluated using the mean absolute percentage error (MAPE); the parameters were calculated on the original experimental scale as $\mathrm{MAPE}\;\left(\%\right)=\;\frac{100}{n}\sum |\left(x_{exp}-x_{pre}\right)/x_{exp}|$, where *n* is the number of experimental observations and the mean absolute error (MAE). Higher R^2^ together with lower MAPE and MAE were considered indicative of better agreement between experimental and model-predicted values.

### 2.13. Statistical Analysis

Data is presented as mean ± standard deviation of technical replicates obtained from the same homogenized sample for each specific treatment-curing cycle. Differences among treatments within each curing cycle were evaluated by one-way analysis of variance (ANOVA), followed by Tukey’s multiple comparison test at a significance level of 95% (*p* ≤ 0.05). Statistical analyses were performed using Minitab 14 (Minitab, Inc., State College, PA, USA). Because the replicates originated from the same pooled sample, they represent analytical rather than independent biological replication.

## 3. Results and Discussion

### 3.1. Water Content Lost During Vanilla Curing

**Figure 1:**
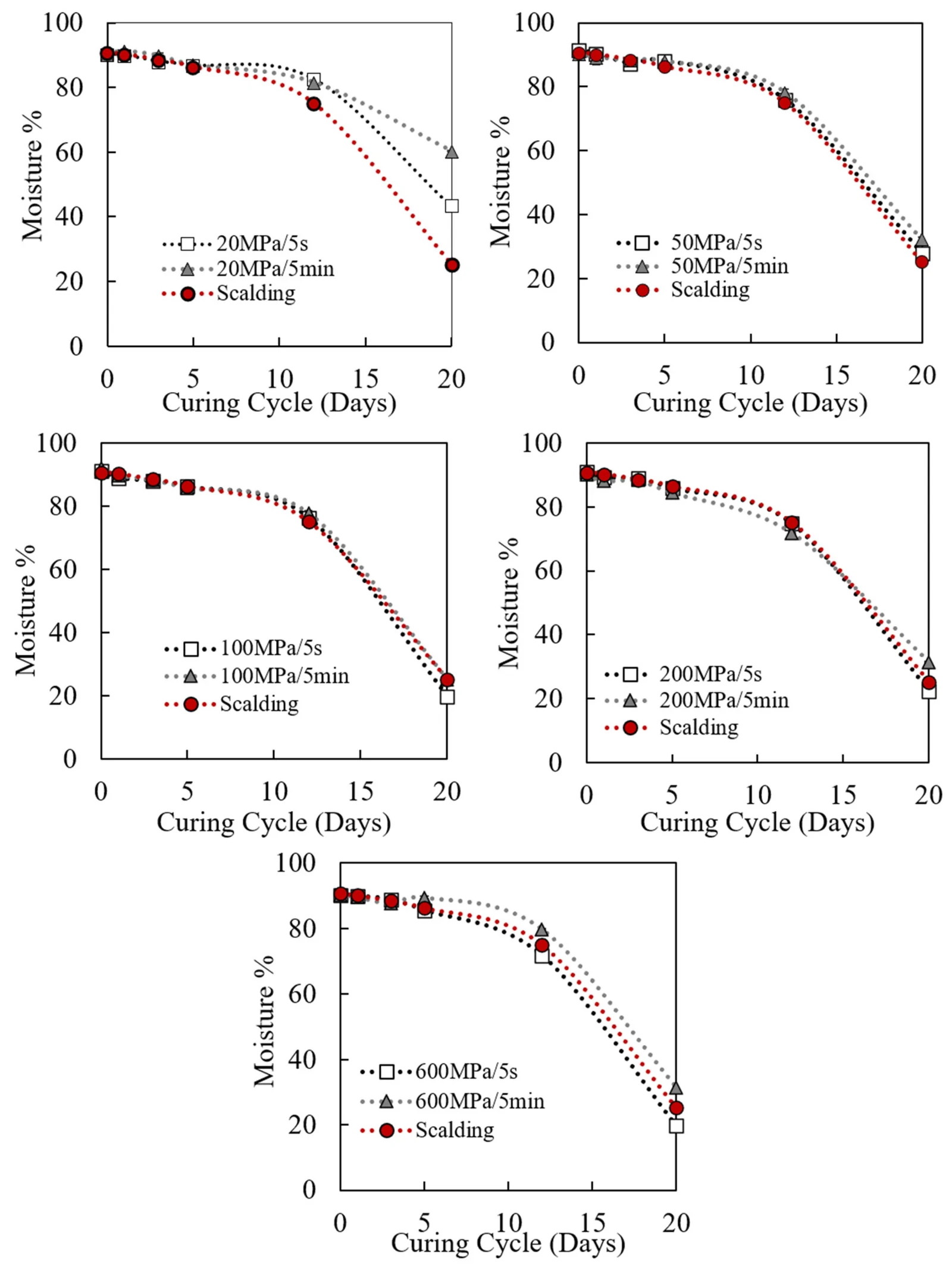
Moisture content (% wet basis) of vanilla beans during curing cycles 0–20 under different high hydrostatic pressure conditions (20, 50, 100, 200 and 600 MPa for 5 s or 5 min) compared with scalding. Error bars represent the standard deviation; when not visible, they are smaller than the data-point symbols.

The initial moisture content of green vanilla pods was approximately 92% (wet basis), consistent with values reported for fresh *Vanilla planifolia* beans [4,5]. Following the killing stage (cycle 0), moisture contents remained similar among HHP-treated samples and scalded beans, between 90 and 92%. This indicates that neither thermal scalding nor HHP induced immediate dehydration (Figure 1). During early stages of curing (cycles 1 to 5), all treatments exhibited a gradual decrease in moisture content to approximately 85–90%, driven by environmental drying conditions rather than the killing method itself [5,7]. However, distinct variations among treatments emerged during later curing stages. By cycle 12, scalded beans reached a moisture content of 75.1%, whereas those subjected to the lowest pressure levels (20 MPa/5 s–5 min) retained higher moisture content (81.7–82.6%). This delayed dehydration may be attributed to lower structural damage and preserved cellular compartmentalization, which enhanced water retention [28].

By the end of curing (cycle 20), scalded beans reached a moisture content of 25.2%, which is characteristic of traditionally cured vanilla [4]. In contrast, HHP-treated pods exhibited distinct behaviors depending on both pressure level and processing duration. Short-time HHP treatments (5 s) resulted in final moisture contents comparable to scalding (19.7–27.8%). Conversely, prolonged HHP treatments (5 min), particularly at low pressure (20 MPa), reduced moisture loss throughout curing, resulting in a final moisture content of 60.4%. A similar pressure-dependent moisture response was reported in mango by Hu et al. (2021) [29], who observed higher moisture retention after harvest when applying low pressures (20–40 MPa), whereas greater moisture loss occurred at higher pressure levels (80 MPa). Those authors suggested that HHP decreases the degree of pectin esterification, thereby increasing water-binding capacity within plant tissues [29]. Consequently, HHP may modify cell-wall structure, water-binding properties, and membrane permeability, potentially contributing to the dehydration differences observed among treatments during vanilla curing [30]. The higher moisture retained under the 20 MPa/5 min condition may subsequently influence downstream enzymatic responses and vanillin formation during later curing stages. However, because the slower dehydration itself may result from the pressure–time treatment, moisture status likely represents an intermediate component of the overall HHP response rather than an independent factor. Since neither microstructure nor pectin characteristics were directly evaluated in this study, these mechanisms remain speculative. Future research should include direct microstructural characterization and analysis of pectin content and degree of esterification to clarify their contribution to moisture retention during vanilla curing [30].

The Weibull parameters were consistent with the experimental moisture-loss profiles. The 20 MPa/5 min treatment showed the highest scale parameter (λ = 28.77 days), indicating a slower characteristic dehydration rate compared to the other treatments, which displayed λ values of approximately 18–22 days (Table 2). This parameter trend directly aligns with the substantially higher moisture content retained by the 20 MPa/5 min samples at cycle 20. Additionally, the shape parameter (k) varied across treatments, reflecting distinct differences in the geometric curvature of their respective dehydration profiles. Overall, the high R^2^ values and low MAPE values supported the suitability of the Weibull model for describing moisture loss during vanilla curing.

### 3.2. β-Glucosidase Activity

**Figure 2:**
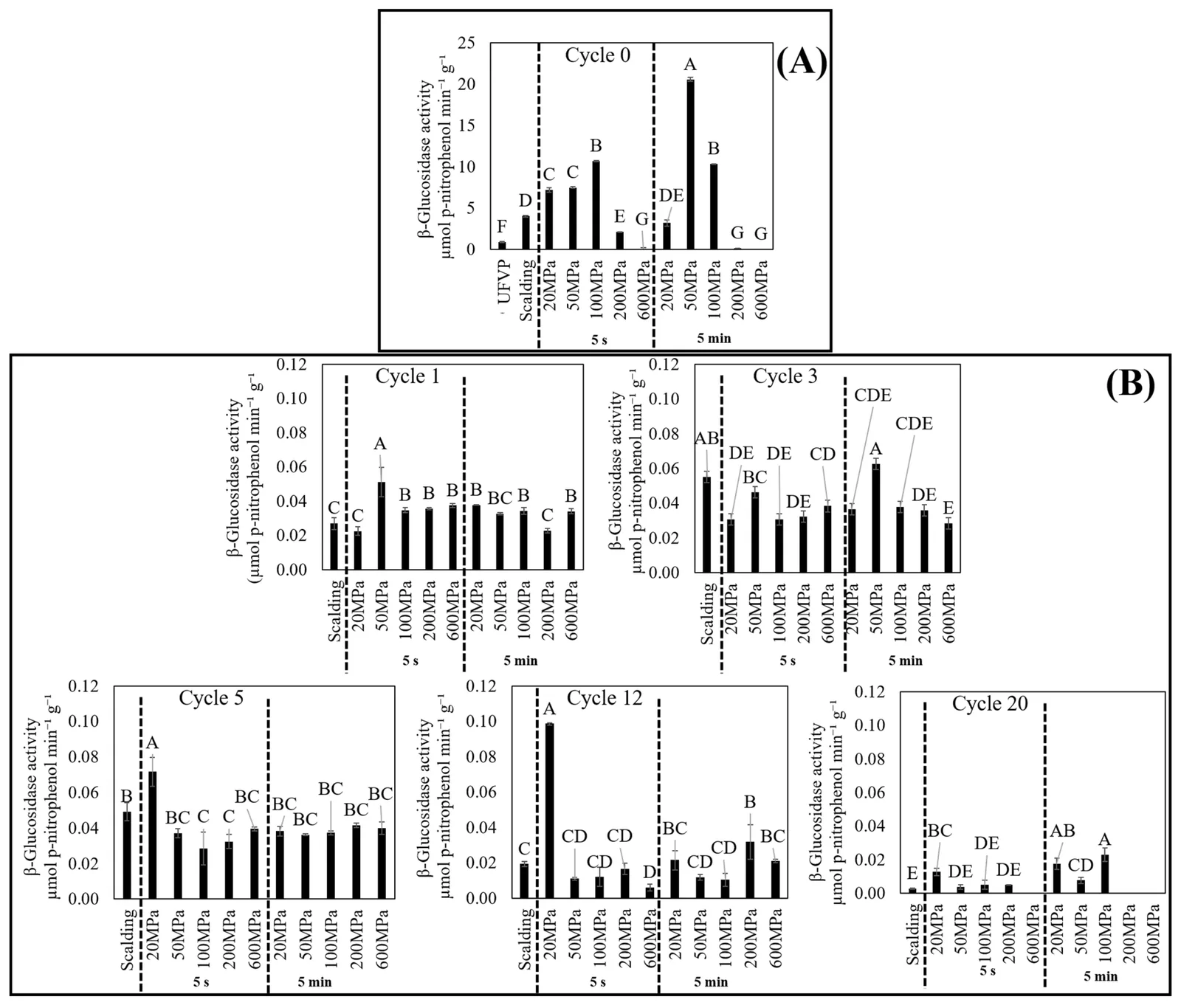
β-Glucosidase activity (µmol p-nitrophenol min^−1^ g^−1^ db) of vanilla beans subjected to HHP treatments (20, 50, 100, 200, and 600 MPa for 5 s or 5 min) and scalding treatment. (**A**) β-Glucosidase activity immediately after the killing treatment (cycle 0), including untreated fresh vanilla pods (UFVP). (**B**) β-Glucosidase activity during subsequent curing cycles (1, 3, 5, 12, and 20). Different *y*-axis scales were used for cycle 0 and subsequent curing cycles because of the higher enzyme activity at cycle 0. Error bars represent the standard deviation. Different letters within the same cycle indicate significant differences according to Tukey’s test (*p* ≤ 0.05).

As shown in Figure 2, the β-glucosidase activity for UFVP was low (0.90 µmol min^−1^ g^−1^ db), which is consistent with the strict compartmentalization of the enzyme and its glycosylated substrates within the intact tissues prior to killing and curing [7,10,31]. β-glucosidase activity was up to 20.6 µmol min^−1^ g^−1^ db, consistent with values previously reported by Odoux et al. [8], which ranged from 2 to 55 µmol min^−1^ g^−1^ db.

Immediately after killing (cycle 0), most treatments caused an increase in β-glucosidase activity, suggesting effective disruption of cellular integrity and enhanced enzyme–substrate contact. Scalded beans reached an activity of 4.07 µmol min^−1^ g^−1^ db, whereas HHP-treated beans exhibited significantly higher activities, particularly at moderate pressures. Treatments between 20 and 100 MPa applied for 5 s resulted in activities ranging from 7.22 to 10.71 µmol min^−1^, g^−1^ db, while the highest activity was observed at 50 MPa for 5 min (20.6 µmol min^−1^ g^−1^ db).

These results indicate that HHP-assisted killing enhances enzyme accessibility while preventing the severe enzymatic damage commonly associated with thermal inactivation, a problem frequently reported during high-temperature scalding in vanilla processing [8,9,10].

However, not all HHP treatments were equally effective. Higher pressures (≥200 MPa) decreased β-glucosidase activity, particularly after 5 min duration treatments. This decrease may reflect pressure-induced enzyme inactivation, reduced extractability, or changes in tissue structure, phenomena widely described for hydrolytic enzymes exposed to intense hydrostatic pressure [32,33]. During subsequent curing cycles, β-glucosidase activity declined sharply in all treatments, reaching very low values by cycle 5 and becoming nearly negligible by cycle 20. This progressive decrease may be associated with advancing tissue dehydration, decreased enzyme stability or extractability and general decline in metabolic activity as curing progresses [4,7]. Overall, these results demonstrate that HHP-assisted killing, particularly at moderate pressures and short exposure times, enhances early β-glucosidase activity relative to conventional scalding, potentially favoring vanillin release during the initial stages of curing.

### 3.3. PAL Activity

**Figure 3:**
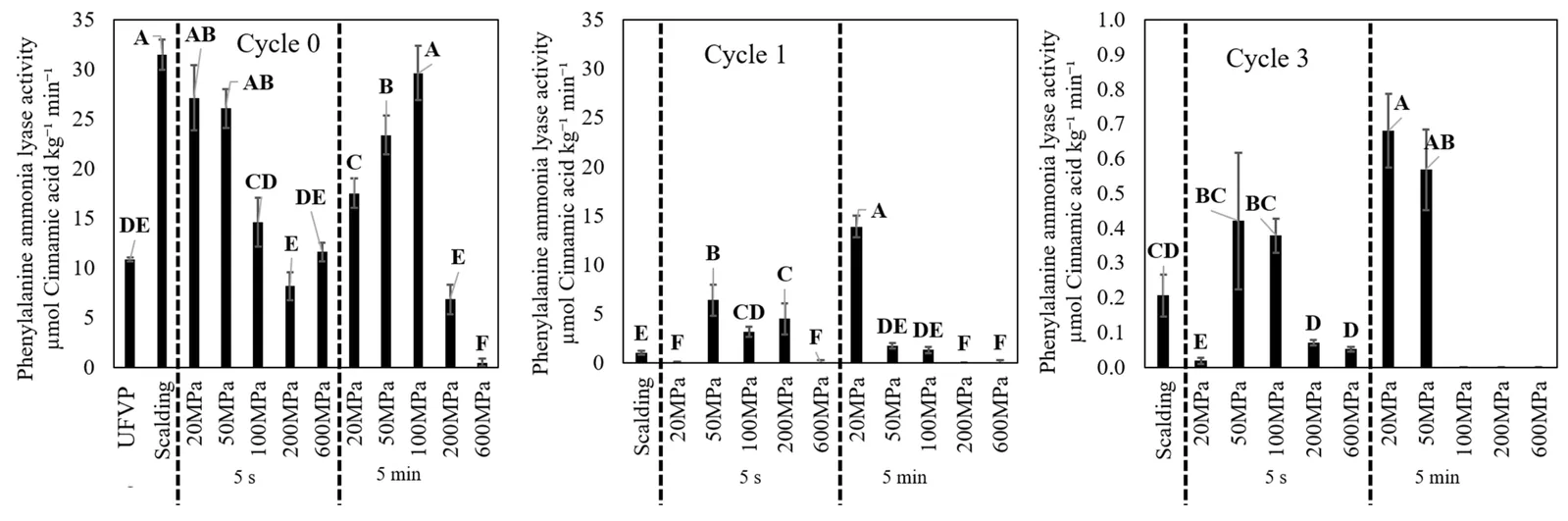
Phenylalanine ammonia lyase activity (µmol trans-cinnamic acid min^−1^ kg^−1^ db) for untreated fresh vanilla pods (UFVP), vanilla beans subjected to HHP treatments (20, 50, 100, 200, and 600 MPa for 5 s or 5 min) or thermal scalding, during the early curing stages (cycles 0, 1, and 3). Error bars represent the standard deviation of replicates. Different letters within the same cycle indicate significant differences according to Tukey’s test (*p* ≤ 0.05). No detectable PAL activity was observed in any treatment from cycles 5 to 20.

Untreated green vanilla had a PAL activity level of 11.20 µmol min^−1^ kg^−1^ db. Following the killing stage and before the beginning of the curing cycles, PAL activity increased markedly in most treatments (Figure 3), reaching 32.43 µmol min^−1^ kg^−1^ db in scalded beans and comparable values (*p* > 0.05) in several HHP-treated samples, particularly at moderate pressures (20–50 MPa for 5 s and 100 MPa for 5 min). PAL is widely recognized as a stress-responsive enzyme whose activity can be affected by mechanical damage, heat, pressure, and other abiotic stimuli [17,18,34]. Therefore, the observed increase may be associated with a physiological response to the killing treatments. Additionally, direct physicochemical effects of pressure on enzyme structure and tissue integrity may also contribute to higher apparent activity. Moderate pressure can induce conformational changes that favor enzyme functionality [15], while cellular disruption may facilitate PAL release during extraction [14]. Nevertheless, the present results do not allow these mechanisms to be distinguished, and further studies evaluating signaling molecules such as reactive oxygen species (ROS) and ethylene would be required to confirm the involvement of stress-induced phenylpropanoid metabolism [17,35,36].

Similar increases in PAL activity have been previously reported in different plant tissues subjected to HHP. For instance, a study in carrots subjected at 60–100 MPa associated a 155% increase in total phenolics with a 380% rise in PAL activity [35]. A similar relationship was reported in potato suspension culture, where a 100 MPa application yielded up to a 456% increment in total polyphenols associated with a 199% increase in PAL activity [37]. In the present study, after the initial measurement, PAL activity started to decrease rapidly, with a marked reduction by cycle 1, followed by a further decrease by cycle 3. Detectable PAL activity remained in some treatments at cycle 3 (except for 100, 200 and 600 MPa/5 min), whereas no activity was detected in any treatment from cycles 5 to 20. This transient activation is consistent with the nature of stress-induced PAL activity and the rapid metabolic shutdown associated with tissue dehydration and senescence during postharvest processing [17,19].

Notably, low- and moderate-pressure treatments, such as 50, 100, and 200 MPa for a duration of 5 s and 20–50 MPa for a duration of 5 min, resulted in higher PAL activity during curing cycle 1, reaching values more than 13 times higher than those for scalded beans (20 MPa/5 s). This response suggests milder physiological stress and a delayed decline in enzymatic activity compared with conventional scalding. Increased PAL activity has been associated with the activation of phenylpropanoid metabolism in plant tissues subjected to abiotic stress [17,19]. Therefore, a similar response may have contributed to the changes observed here. Although this mechanism was not directly evaluated in the present study, PAL activity was clearly restricted to the early stages of curing; thus, it should be interpreted in relation to its role in the phenylpropanoid pathway rather than as a direct mechanism of vanillin formation. In contrast, the hydrolysis of glucovanillin to vanillin is mediated by β-glucosidase.

Together, these observations indicate that although PAL and β-glucosidase drive critical biochemical events during the early stages of curing, the subsequent development of the characteristic properties of cured vanilla cannot be attributed exclusively to these enzymes. Other enzymatic systems and non-enzymatic transformations likely contribute to the biochemical changes occurring during the later stages of curing.

### 3.4. Total Phenolic Content (TPC)

The Folin–Ciocalteu method is a reliable and very useful tool for estimating total phenolic content in a complex matrix like vanilla. However, because this assay relies on the reduction of the phenol group (-OH), it is not fully selective and also reacts with glycosylated phenolic compounds, such as glucovanillin (GV). Consequently, results represent the total phenolic content, including both glycosylated and non-glycosylated compounds [38,39].

**Figure 4:**
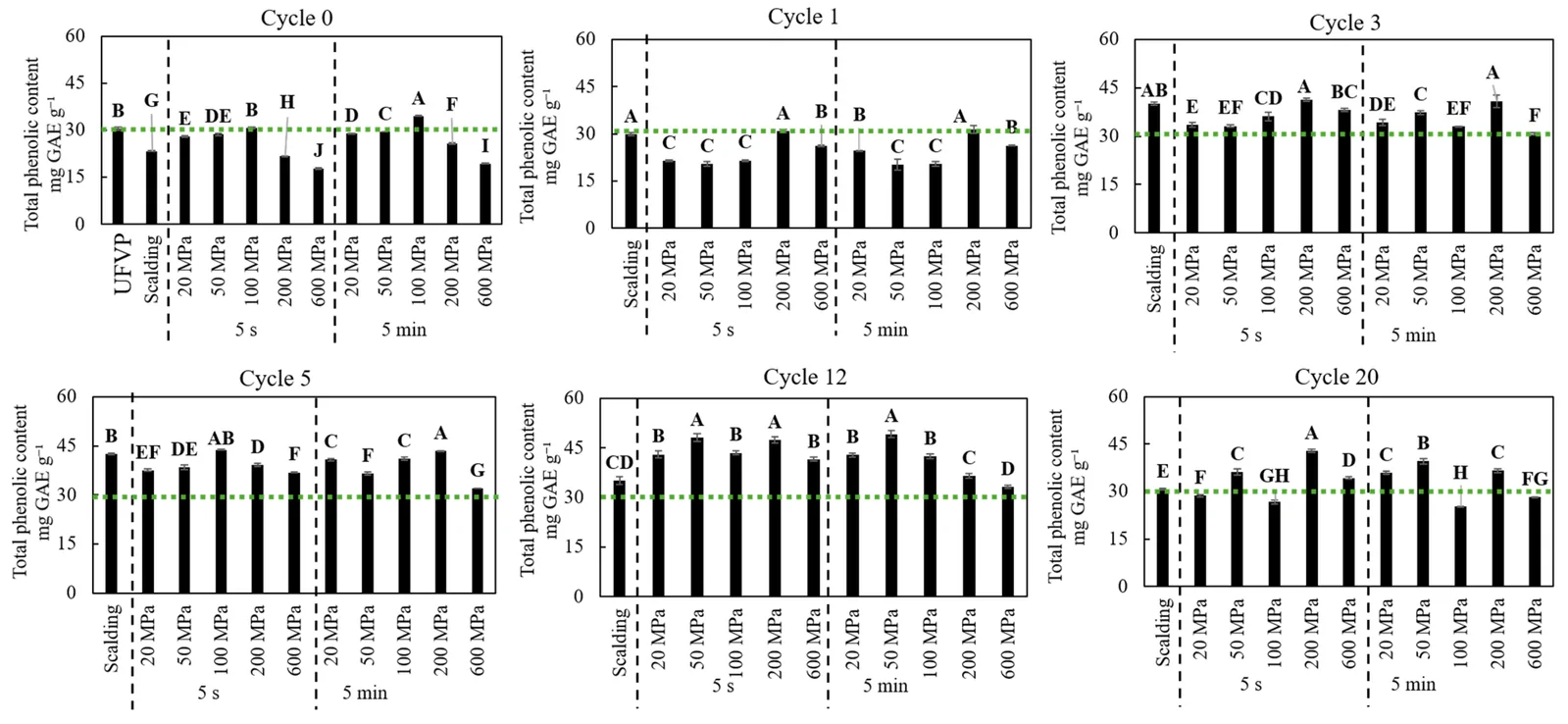
Total phenolic content (mg GAE g^−1^ db) of vanilla beans subjected to HHP treatments (20, 50, 100, 200, and 600 MPa for 5 s or 5 min), or conventional thermal scalding throughout the curing cycles. The horizontal dotted line represents the total phenolic content of untreated fresh vanilla pods (UFVP; 30.5 mg GAE g^−1^ db). Error bars represent the standard deviation of replicates. Different letters within the same cycle indicate significant differences according to Tukey’s test (*p* ≤ 0.05).

TPC of green vanilla beans was 30.5 mg GAE g^−1^ dry matter (Figure 4), a value consistent with previous reports for fresh *Vanilla planifolia* pods prior to curing [4,5]. Following the killing stage in cycle 0, the phenolic content varied markedly depending on the treatment applied. Scalded beans exhibited a noticeable reduction in phenolic content (23.3 mg GAE g^−1^), which may be attributed to thermal degradation, leaching into the scalding water, or heat-induced oxidation [9,38,40]. In contrast, HHP-treated beans generally maintained phenolic levels closer to those of UFVP, suggesting that non-thermal cell disruption preserves phenolic integrity during the killing stage, except for the 200 and 600 MPa treatments for both times after UFVP [33].

During the curing process after cycle 3, total phenolic content increased progressively in all treatments, reaching maximum values between cycles 5 and 12. This accumulation has been widely associated with stress-induced activation of the phenylpropanoid pathway during postharvest handling, as well as the early activities of PAL and β-glucosidase [7,17,19,35]. HHP-treated beans, particularly those subjected to moderate pressures (50–200 MPa), exhibited significantly higher phenolic contents compared to scalded beans. with maximum values up to 49.1 mg GAE g^−1^ for the 50 MPa/5 min treatment at cycle 12; these values were statistically similar to those obtained for the 50 MPa/5 s and 200 MPa/5 s treatments at the same cycle. Similar increases in phenolic content have been reported in plant tissues exposed to moderate abiotic stress and have been associated with changes in PAL activity and phenylpropanoid metabolism [13,18].

At later curing stages (cycle 20), a slight decline in phenolic content was observed across treatments, likely due to oxidative reactions, polymerization, or the binding of phenolics to macromolecules during prolonged drying and conditioning [7,40]. Overall, these results indicate that HHP-assisted killing enhances phenolic accumulation during curing more effectively than traditional scalding, thereby promoting greater phenolic retention and potentially augmenting the final antioxidant properties.

### 3.5. Glucovanillin and Vanillin Content

**Figure 5:**
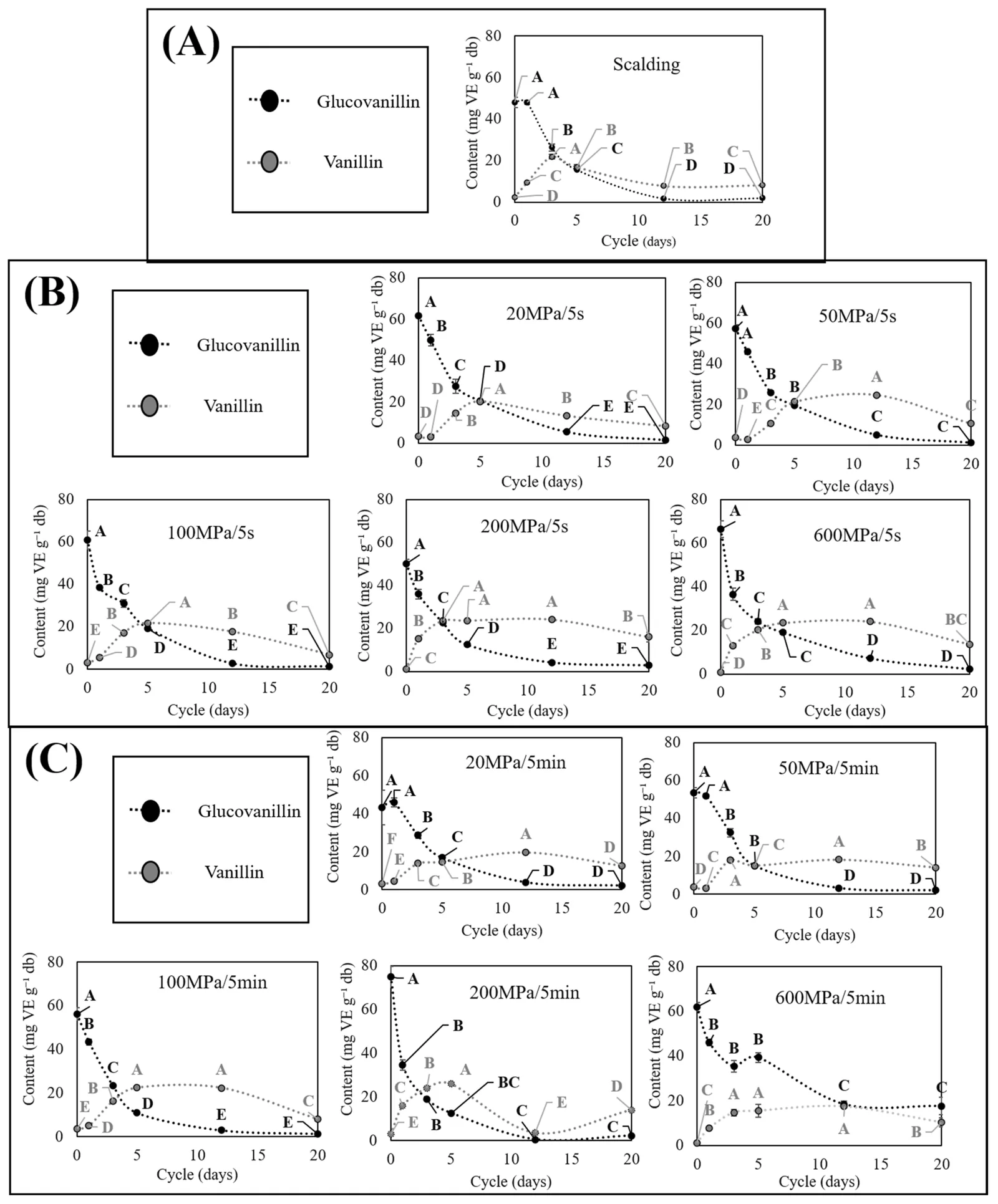
Glucovanillin and vanillin contents (mg VE g^−1^ db) during curing of vanilla beans subjected to (**A**) conventional thermal scalding, (**B**) HHP treatments at 20, 50, 100, 200, and 600 MPa for 5 s, and (**C**) HHP treatments at 20, 50, 100, 200, and 600 MPa for 5 min. Error bars represent the standard deviation; when not visible, they are smaller than the corresponding data-point symbols. Black letters correspond to glucovanillin, whereas gray letters correspond to vanillin. Different letters within the same cycle and compound indicate significant differences according to Tukey’s test (*p* ≤ 0.05).

Glucovanillin (GV) was the predominant phenolic compound detected in untreated fresh vanilla pods (47.03 ± 2.00 mg g^−1^ db), consistent with its role as the main storage and precursor form of vanillin in *Vanilla planifolia* [7,8]. Immediately after the killing treatment, several HHP conditions showed higher measured GV concentrations than both untreated and scalded samples (Figure 5), including 20 MPa/5 s (61.76 mg g^−1^ db), 100 MPa/5 s (60.83 mg g^−1^ db), and 600 MPa/5 min (61.81 mg g^−1^ db). The highest concentration was observed at 200 MPa/5 min (75.08 mg g^−1^ db), followed by 600 MPa/5 s (66.46 mg g^−1^ db); according to Tukey’s test, these two treatments were not significantly different from each other (*p* > 0.05). Nevertheless, these elevated concentrations should not be interpreted as evidence of instantaneous de novo glucovanillin formation during HHP treatment. Instead, pressure-induced disruption of cellular structures and decompartmentalization may increase glucovanillin accessibility and extraction efficiency, resulting in a greater amount of extractable GV being recovered [32,33]. Similar effects have been reported for non-thermal technologies applied to phenolic-rich plant matrices [13].

The decrease in GV was adequately described by the pseudo-first-order kinetic model (Table 3), with maximum *k* values of 0.43 day^−1^ from 200 MPa/5 min and 0.40 day^−1^ from 50 MPa/5 min, which were not significantly different (*p* > 0.05). The other treatments exhibited k values between 0.29 and 0.36 day^−1^, except for the 600 MPa/5 min condition, which showed the lowest rate (0.16 day^−1^). The main findings from this study indicate that the effect of HHP on glucovanillin loss during curing depends on the combination of pressure level and treatment duration rather than a simple pressure-dependent trend. Model performance was evaluated considering R^2^ together with the mean absolute percentage error (MAPE) and mean absolute error (MAE). R^2^ values were ≥0.82, while MAPE and MAE values ranged from 4.72 to 23.67% and 1.00 to 6.9 mg g^−1,^ respectively. The combined evaluation of these parameters revealed variations in model performance across treatments. For example, the 200 MPa/5 s treatment showed an exceptionally high R^2^ (0.99) along with the lowest MAPE (4.72%) and MAE (1.00 mg g^−1^), indicating an excellent fit between the experimental and predicted glucovanillin concentrations. However, some treatments presented high MAPE values mainly attributed to glucovanillin concentrations approaching near-zero values during the final curing stages, where small absolute deviations can result in relatively large percentage errors. Therefore, MAE was included as a complementary metric to evaluate model fit in absolute concentration units.

Vanillin content in UFVP at cycle 0 was 3.21 mg g^−1^ db. Similar results were obtained in most HHP treatments, except for 200 MPa/5 s, 600 MPa/5 s, and 600 MPa/5 min with 1.04, 0.85, and 1.08 mg g^−1^ db, respectively. Vanillin accumulation reached the maximum values between cycles 3 and 12, depending on the treatment (Figure 5). Compared with scalding, most HHP treatments resulted in higher vanillin concentrations, particularly after cycle 5, despite the progressive decline in vanillin observed at later curing stages. Notable increases included 80% for the 200 MPa/5 min treatment at cycle 5 and 97% for the 200 MPa/5 s treatment at cycle 20. However, the greatest relative increases were observed at cycle 12 in the 50 MPa/5 s and 200 MPa/5 s treatments, which showed increments of 217.9% and 211.2%, respectively, corresponding to vanillin concentrations of 24.6 and 24.1 mg g^−1^ db. The occurrence of the maximum vanillin concentrations during the intermediate curing stages was not exclusive to HHP-treated beans. Similar temporal patterns have been reported during conventional vanilla curing, where vanillin accumulates during the early-to-intermediate stages and subsequently declines as curing progresses [41,42]. Therefore, HHP appears to modulate the magnitude of vanillin accumulation rather than fundamentally altering the characteristic temporal pattern of vanillin formation during curing.

The concurrent decrease in glucovanillin and increase in vanillin during the early and intermediate curing stages are consistent with β-glucosidase-mediated hydrolysis of GV [7,9], although a quantitative conversion yield cannot be inferred from the present data. The greater vanillin accumulation observed under selected HHP conditions may be related to the higher β-glucosidase activity measured during the early curing stages and may also reflect pressure-induced tissue disruption and decompartmentalization, which could facilitate enzyme–substrate contact. In contrast, thermal scalding has been reported to partially or fully inactivate β-glucosidase [9,10].

At the final curing stage (cycle 20), vanillin levels decreased slightly in most treatments, after approximately six curing cycles, the vanillin concentration began to decline gradually. Although the mechanisms responsible for this reduction are not fully understood, it has been suggested that vanillin may undergo volatilization, oxidation, or transformation into secondary aroma compounds during prolonged conditioning [4,12,41,42]. Collectively, these results indicate that selected HHP conditions can favorably modulate glucovanillin hydrolysis and vanillin formation compared with traditional scalding.

### 3.6. Antioxidant Capacity

**Figure 6:**
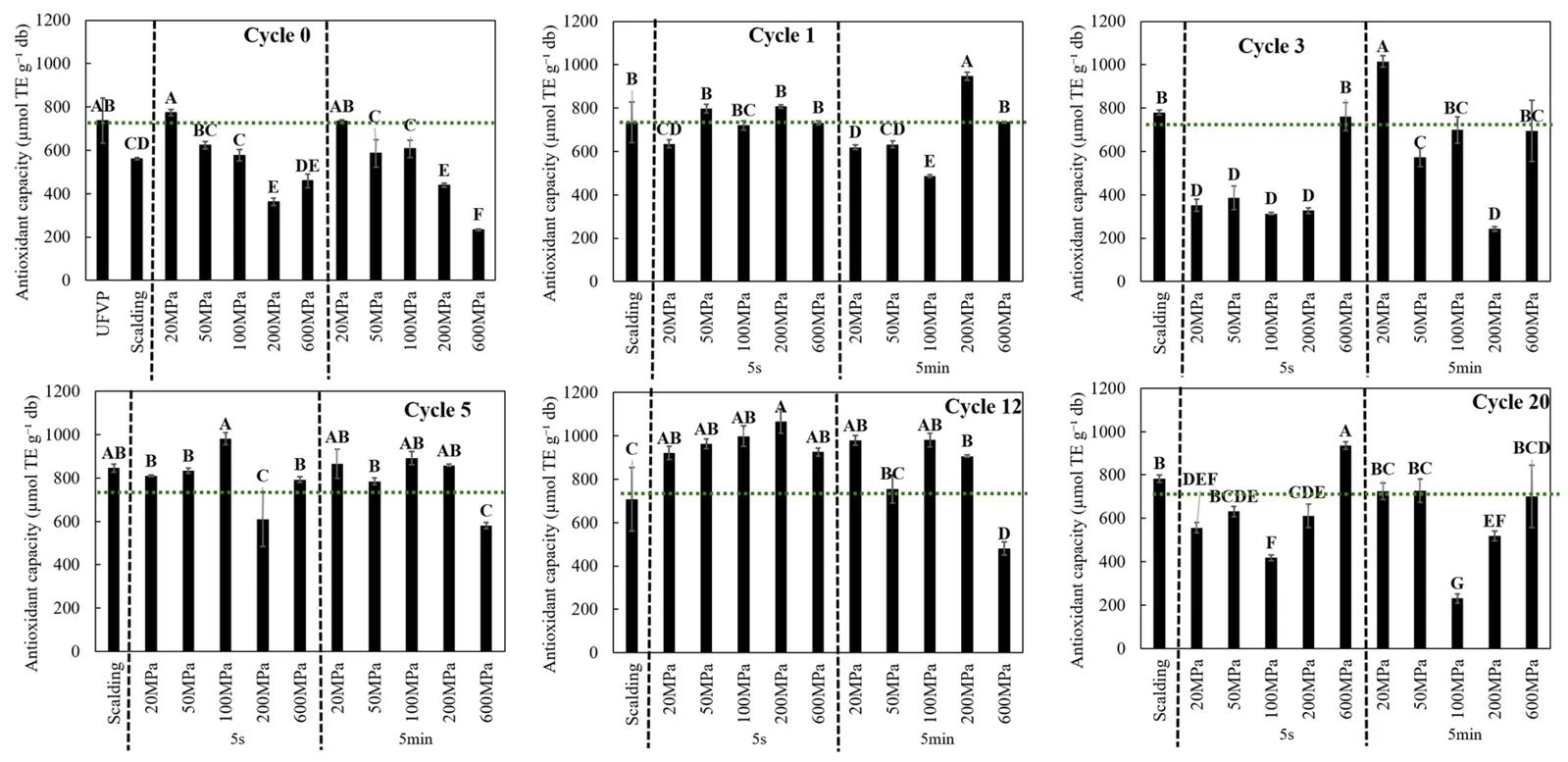
Antioxidant capacity (µmol TE g^−1^ db) of untreated fresh vanilla pods (UFVP), conventionally scalded vanilla beans, and vanilla beans subjected to HHP treatments (20, 50, 100, 200, and 600 MPa for 5 s or 5 min), during curing cycles 0–20. The horizontal dotted line represents the antioxidant capacity of untreated fresh vanilla pods (UFVP; 737.16 µmol TE g^−1^ db). Error bars represent the standard deviation. Different letters within the same cycle indicate significant differences according to Tukey’s test (*p* ≤ 0.05).

The antioxidant capacity of untreated fresh vanilla pods (UFVP) was 737.2 ± 105.2 µmol TE g^−1^ db (Figure 6). For comparison, the USDA Database for the Oxygen Radical Absorbance Capacity (ORAC) of Selected Foods reported a Total-ORAC value of 1224 µmol TE g^−1^ db for dried vanilla beans [43]. However, the database value serves primarily as a baseline reference, since variations in extraction procedure, sample origin, curing stage, and analytical conditions strongly influence the measured antioxidant capacity.

Immediately after the killing treatment (cycle 0), antioxidant capacity varied considerably among treatments, ranging from approximately 233.6 to 774.2 µmol TE g^−1^ db. The highest mean value was observed for the 20 MPa/5 s treatment (774.2 µmol TE g^−1^ db), whereas the 20 MPa/5 min treatment (734.7 µmol TE g^−1^ db) showed an antioxidant capacity comparable in magnitude to that of untreated fresh vanilla pods (UFVP; 737.2 µmol TE g^−1^ db). During subsequent curing cycles, ORAC values showed a treatment- and cycle-dependent response rather than a uniform effect of HHP. At cycle 1, the highest mean value was observed for 200 MPa/5 min (947.9 µmol TE g^−1^ db), whereas at cycle 3 the highest antioxidant capacity was obtained for 20 MPa/5 min (1015.6 µmol TE g^−1^ db). Subsequently, at cycle 5, 100 MPa/5 s reached 982.0 µmol TE g^−1^ db.

A notable antioxidant response was observed at cycle 12, when most HHP treatments showed ORAC values between approximately 869 and 1067 µmol TE g^−1^ db. The highest value overall was observed for 200 MPa/5 s (1066.6 ± 53.1 µmol TE g^−1^ db), which was approximately 51% higher than the mean value obtained for the scalded treatment at the same cycle (706.4 ± 146.5 µmol TE g^−1^ db). A similar increase in antioxidant capacity has been reported in plant tissues exposed to moderate abiotic stress and are widely associated with changes in phenylpropanoid metabolism [19,44]. While a similar response may have occurred here, the present results do not directly demonstrate activation of the phenylpropanoid pathway or de novo biosynthesis of antioxidant compounds.

Relatively high ORAC values during intermediate curing stages coincided with the period in which elevated total phenolic contents were also observed. This temporal association is consistent with the recognized contribution of phenolic compounds to the antioxidant capacity of plant extracts [45,46,47]. However, because a direct correlation analysis between total phenolic content and ORAC was not performed, this relationship should be interpreted cautiously and does not demonstrate a direct causal association.

At later curing stages (cycle 20), a slight decline or stabilization in antioxidant capacity was observed across all treatments. Changes during the later stages of curing may be associated with oxidation, polymerization, or other transformations of phenolic compounds during prolonged drying and conditioning [4,11,12]. Overall, the ORAC response was dependent on both HHP treatment conditions and curing stage. Under selected HHP conditions, particularly during intermediate curing stages, the antioxidant capacity surpassed that observed after conventional scalding.

## 4. Conclusions

This study demonstrates the potential of high hydrostatic pressure as a non-thermal alternative to conventional scalding for the killing stage of *Vanilla planifolia* curing. Rather than identifying a single all-encompassing optimal processing condition, the results revealed distinct biochemical and physical responses depending on the pressure level and exposure time. Moderate pressure, such as 50 MPa for 5 min, provided the most balanced response. This specific condition preserved higher β-glucosidase activity during curing, which correlated with high phenolic accumulation, elevated antioxidant capacity, and the retention of superior vanillin levels throughout curing compared to scalding. In contrast, 200 MPa for 5 s was associated with glucovanillin loss, vanillin accumulation during the early curing cycles, and faster moisture loss. Consequently, this latter condition represents a highly effective strategy for promoting dehydration while maintaining favorable glucovanillin and vanillin profiles during curing.

These findings indicate that HHP may provide different processing advantages depending on the specific industrial objective, whether maximizing vanillin content or improving process efficiency to reduce curing time. However, several limitations of the present study should be noted. First, samples from each treatment and curing cycle were analyzed from a single homogenized batch of vanilla with technical replicates rather than independent biological replicates, which restrains the evaluation of true biological variability. Second, volatile aroma compounds and sensory attributes were not evaluated; therefore, the observed changes in vanillin and other biochemical parameters cannot be directly interpreted as improvements in the final aroma or sensory quality of cured pods. Furthermore, the proposed mechanisms associated with stress-induced enzymatic responses were not directly confirmed through the measurement of signaling molecules. Future studies should therefore include independent biological replicates, volatile aroma profiling, and sensory evaluation while validating the most promising HHP conditions under laboratory and industrial-scale curing conditions.

## Figures and Tables

**Table 1:** High hydrostatic pressure treatment conditions, including average chamber temperature and come-up time.

Treatment	Average Temperature (°C)	Come-up Time (s)
20 MPa/5 s	24.8 ± 0.14	10 to 15
20 MPa/5 min	23.1 ± 0.14	10 to 15
50 MPa/5 s	25.1 ± 0.08	15 to 20
50 MPa/5 min	23.5 ± 0.06	15 to 20
100 MPa/5 s	26.4 ± 0.17	25 to 30
100 MPa/5 min	24.5 ± 0.05	25 to 30
200 MPa/5 s	27.9 ± 0.28	40 to 45
200 MPa/5 min	26.5 ± 0.21	40 to 45
600 MPa/5 s	38.9 ± 0.43	85 to 90
600 MPa/5 min	34.5 ± 1.07	85 to 90

Come-up time (CUT) corresponds to the time required for the system to reach the target pressure. Treatment time (5 s or 5 min) refers to the holding time at the target pressure and does not include CUT. Average temperature corresponds to the median value of the recorded temperatures every 5 s during holding time.

**Table 2:** Weibull model parameters for moisture loss in vanilla pods subjected to scalding or HHP treatments.

Treatment	λ (Days)	k	R^2^	MAPE (%)
Scalding	18.718	3.665	0.995	1.400
20 MPa/5 s	21.579	4.055	0.991	1.145
50 MPa/5 s	19.065	3.575	0.992	1.625
100 MPa/5 s	18.059	4.140	0.990	2.139
200 MPa/5 s	18.321	3.777	0.993	1.745
600 MPa/5 s	17.851	3.633	0.996	1.319
20 MPa/5 min	28.769	2.521	0.991	0.817
50 MPa/5 min	19.838	3.827	0.997	0.999
100 MPa/5 min	18.724	3.863	0.983	2.573
200 MPa/5 min	19.739	2.845	0.992	1.846
600 MPa/5 min	19.751	4.193	0.997	0.732

The Weibull model, expressed as $M_{t}/M_{0}={\mathrm{e}}^{-{\left(t,/,\lambda \right)}^{k}}$, where M_t_ and M_0_ represent the moisture content (% wet basis) at the time of curing and at day 0, respectively; *t* is the curing time (days); *λ* is the scale parameter (days); and *k* is the dimensionless shape parameter. The coefficient of determination (R^2^) was calculated on the original moisture-content scale as $R^{2}=1-\sum {\left(M_{exp}-M_{pred}\right)}^{2}/\sum {\left(M_{exp}-{\bar{M}}_{exp\;}\right)}^{2}$. Mean absolute percentage error (MAPE) and mean absolute error (MAE) were used as complementary goodness-of-fit metrics to evaluate model adequacy.

**Table 3:** Pseudo-first-order kinetic parameters for glucovanillin loss during vanilla curing.

Glucovanillin				
Treatment	k (Day^−1^)	R^2^	MAPE (%)	MAE (mg g^−1^)
Scalding	0.32 ^bc^	0.99	16.56	5.06
20 MPa/5 s	0.32 ^bc^	0.94	17.48	2.97
50 MPa/5 s	0.31 ^bc^	0.92	17.61	2.83
100 MPa/5 s	0.36 ^ab^	0.97	18.87	4.21
200 MPa/5 s	0.29 ^c^	0.99	4.72	1.00
600 MPa/5 s	0.33 ^bc^	0.89	20.38	4.16
20 MPa/5 min	0.30 ^bc^	0.99	22.39	6.14
50 MPa/5 min	0.40 ^a^	0.97	23.67	6.90
100 MPa/5 min	0.32 ^bc^	0.99	6.67	1.06
200 MPa/5 min	0.43 ^a^	0.99	16.35	4.61
600 MPa/5 min	0.16 ^d^	0.82	10.26	4.34

Pseudo-first-order model was expressed as $\ln\left[\left(C-C_{F}\right),/,\left(C_{i}-C_{F}\right)\right]=-kt$, where C is the glucovanillin concentration at a certain curing cycle, Ci and Cf are the mean initial (from cycle 0) and final (from cycle 20) glucovanillin concentration for each treatment, t is the curing time (days) and K is the pseudo-first-order rate constant (days^−1^). K was determined as the negative slope of the linear regression of $\ln\left[\left(C-C_{F}\right),/,\left(C_{i}-C_{F}\right)\right]$ versus the curing time. R^2^ represents the coefficient of determination of this linearized regression. Predicted glucovanillin content were calculated and used to determine the mean absolute percentage error (MAPE %) and the mean absolute error (MAE, mg g^−1^). Different superscript lowercase letters indicate significant differences among k values (*p* ≤ 0.05).

## Data Availability

The original contributions presented in this study are included in the article. Further inquiries can be directed to the corresponding authors.

## Abbreviations

The following abbreviations are used in this manuscript:

CTS	Conventional thermal scalding
HHP	High hydrostatic pressure
CUT	Come-up time
PAL	Phenylalanine ammonia-lyase
TPC	Total phenolic content
RH	Relative humidity
p-NPG	p-Nitrophenyl β-D-glucopyranoside
PVPP	Polyvinylpolypyrrolidone
GV	Glucovanillin
VE	Vanillin equivalents
ROS	Reactive oxygen species
GAE	gallic acid equivalents
ORAC	Oxygen radical absorbance capacity
TE	Trolox equivalents
AAPH	2,2′-Azobis(2-amidinopropane) dihydrochloride
